# Gender-specific association of loneliness and health care use in community-dwelling older adults

**DOI:** 10.1186/s12877-023-04201-9

**Published:** 2023-08-21

**Authors:** Friederike Hildegard Boehlen, Dirk Heider, Dieter Schellberg, Johanna Katharina Hohls, Ben Schöttker, Hermann Brenner, Hans-Christoph Friederich, Hans-Helmut König, Beate Wild

**Affiliations:** 1 Department of General Internal Medicine and Psychosomatics, Medical University Hospital, Im Neuenheimer Feld 410, 69120 Heidelberg, Germany; 2 Department of Health Economics and Health Services Research, University Medical Center Hamburg-Eppendorf, 20251 Hamburg, Germany; 3 Division of Clinical Epidemiology and Aging Research, German Cancer Research Center, 69120 Heidelberg, Germany; 4 Network Aging Research, Heidelberg University, 69115 Heidelberg, Germany

**Keywords:** Loneliness, Gender differences, Health care use, Health care costs, Elderly persons

## Abstract

**Background:**

Loneliness in older adults is common, particularly in women. In this article, gender differences in the association of loneliness and health care use are investigated in a large sample of community-dwelling older adults.

**Methods:**

Data of 2525 persons (ages 55–85 years)—participants of the fourth follow- up (2011–2014) of the ESTHER study- were analyzed. Loneliness and health care use were assessed by study doctors in the course of a home visit. Gender-specific regression models with Gamma-distribution were performed using loneliness as independent variable to predict outpatient health care use, adjusted for demographic variables.

**Results:**

In older women, lonely persons were shown to have significantly more visits to general practitioners and mental health care providers in a three-month period compared to less lonely persons (p = .005). The survey found that outpatient health care use was positively associated with loneliness, multimorbidity, and mental illness in older women but not in older men. Older men had significantly more contact with inpatient care in comparison to women (p = .02).

**Conclusions:**

It is important to consider gender when analyzing inpatient and outpatient health care use in older persons. In older women loneliness is associated with increased use of outpatient services.

## Background

Loneliness in older adults is a challenge - for the health and well-being of the individual as well as for the health care system itself. Although loneliness is a social phenomenon rather than a disease, it appears to have an impact on how and how often persons contact health care providers.

Lonely persons report a 1.3–1.8 higher rate of accessing health care services compared to those who are not lonely [1]. Loneliness is suspected of leading to more contact with emergency departments, more frequent consultations with outpatient care providers, and an increased use of psychotropic drugs; it is also associated with longer hospital stays and increased rates of re-admission [2–4]. In addition, it was found that lonely persons have a remarkably negative perception of their own health status and show a weaker treatment adherence [3]. Loneliness also has a greater, although indirect, impact on health care use when worsening physical and mental well-being become evident, particularly in older people. It is associated with cardiovascular disease and dementia [5], as well as with depression, anxiety, suicidal ideation and a reduced health-related quality of life [6, 7]. During the COVID-19- pandemic loneliness- due to social distancing- has shown to be the main risk factor for depression and anxiety [8]. Vice versa, mental diseases can lead to reduced social contacts; thus, a mutual relationship between social distancing and mental diseases can be assumed. Interestingly, a recent study in an older population showed that self-perceived isolation mediated the relationship between depression symptoms and social disconnectedness [9].

In older adults, loneliness is frequent. In a recent population-based study, the prevalence of loneliness was estimated to be about 10.5%, with a decline across age groups (range: 35–74 years) [10]. Marital status, mental morbidity, and poor current health appeared to be vulnerability factors for loneliness whereas having a post-secondary education was shown to be protective [11]. If and when social networks change and become fragile in the course of the ageing process, the risk for loneliness increases.

Women and men differ in their need to belong to someone, in their perception of loneliness, and in the way they connect with others. While several studies, as well as our own published data, show that loneliness is higher in older women in comparison to men [2, 8, 12], other studies found that older men were somewhat lonelier than women [13]. The onset of a disease often changes the way one perceives one`s social network and increases the need for contact and support. Own previous research showed that loneliness had a greater impact on physical health- related quality of life in older women in comparison with older men [14]. It can therefore be hypothesized that lonely women and lonely men react differently to the occurrence of sudden illness, and that they also differ in their way of addressing health care providers. Women, for instance, were shown to report symptoms more frequently in a medical context, have more frequently complex health care needs and to make greater use of health care services and medication [15–17].

In conclusion, to date few studies have investigated the impact of loneliness and social isolation on health care use. However, studies that focus on gender-specific association between loneliness and consultation-seeking remain scarce [18].

The primary aim of this study was, therefore, to investigate the association of loneliness and health care use, stratified by gender. We hypothesized that in older women the association between loneliness and health care use would be stronger than in older men. Our second aim was to compare the health care use of various outpatient sectors between lonely and less lonely older persons, stratified by gender.

## Methods

### Study sample

The study sample is part of the ESTHER study—a population-based cohort study in Germany (Federal State of Saarland). The aim of this study is to collect and analyze epidemiological data on prevention, early recognition, and optimal treatment of chronic diseases in an older population [19, 20]. The study population (n = 9949 at baseline) was originally recruited between July, 2000, and December, 2002, by general practitioners during a health check-up that is offered to adults (over age 35) in Germany. At baseline the ESTHER study sample was shown to be representative of the general German population regarding chronic diseases and demographic criteria [20]. Follow-ups were conducted 2, 5, 8, 11, 14, and 17 years later and the 20-year follow-up is ongoing. In the fourth follow-up, after 11 years (2011–2014), 4981 persons participated in the ESTHER study; 2761 of these also decided to attend an extensive home visit that was conducted by trained study doctors. The home visit was comprised of the assessment of medical, pharmacological, socio-economic, and psychosocial data as well as functional aspects. This current study is based on cross-sectional data of the ESTHER participants who attended the fourth follow-up, including the additional home visit.

### Measurements

#### Health care use and health care costs

Use of outpatient health care resources (15 possible categories) and inpatient health care were assessed during the home assessment by an established questionnaire [21]. For outpatient health care use participants were asked if and how often they had contacts with providers of the following over the past three months: (1) general medicine (2) gynecology (3) cardiology (4) urology (5) psychiatry (6) neurology (7) orthopedics (8) dental care (9) otolaryngology (10) radiology (11) dermatology (12) psychology (13) ophthalmology (14) emergency medicine (15) alternative medicine (16) physiotherapy (17) other. We calculated the utilization of specific areas of outpatient health care by summing up the listed ambulatory contacts over the past three months for (a) emergency medicine (b) medical specialist care and (c) mental health care. Inpatient health care was assessed by the number of days spent in hospitals, rehabilitation hospitals, and/or psychiatric clinics. In addition, study doctors scanned and listed all medications of the participants and assessed the use of medical supplies, nursing care, and out-of-pocket costs for the previous three months. Health care costs for outpatient and inpatient contact were calculated by using German unit costs for the respective goods and services at 2009 price levels (in €). The assessment of health care use and the method to estimate health care costs were validated in previous studies [22]. For outpatient care, the cost of contact with providers of outpatient medical services was calculated by means of several sources of unit costs: reimbursement schedules (Verband der Ersatzkassen, 2001, 2002, 2007), schedules of fees (Bundesverwaltungsamt, 2011), and calculated costs per contact by specialization. Costs of medical supplies and medication were included.

#### Loneliness

The degree of loneliness was measured by using three items on social isolation and loneliness, derived from the Groningen Frailty Indicator (GFI) [23, 24]. The GFI had been validated in large surveys for the assessment of frailty. Social isolation and loneliness are measured by using three items: “Does the patient sometimes experience an emptiness around him/her?“, “Does the patient sometimes miss people around him/her?”, “Does the patient sometimes feel abandoned?”. The response categories are coded 1 (hardly ever), 2 (some of the time), and 3 (often). We calculated a sum score, with higher scores indicating greater loneliness. A cut-off score of seven and greater was used to categorize the sample into two subgroups. Thus, persons were defined as being ’more lonely’ if they answered at least one of the mentioned questions with “often” and the other questions with “sometimes” [2].

Data on loneliness or mental and physical health were assessed during standardized interviews by trained study doctors.

#### Covariates

The presence of clinically significant symptoms of mental diseases (as a measure of mental health) was coded 0/1 according to the prevalence of depression symptoms or somatization symptoms or generalized anxiety symptoms. Depression severity and severity of generalized anxiety were measured by using the PHQ depression-module (PHQ-9) and the GAD-7 [25, 26]. Somatic symptom severity was measured by an adapted version of the PHQ-15 [27] with 13 questions, including questions about physical pain but excluding questions concerning problems during menstruation or sexual intercourse. Persons were defined as being depressive if they fulfilled (a) the criteria of the PHQ-9 for major depression, (b) minor depression, or (c) if they had a PHQ-9-score > 10 [28]. Persons with a somatic symptom score ≥ 13 were categorized as having somatization disorder in comparison to participants with a score < 13; if they had a GAD-7 score greater than 10 [29], participants were categorized as having generalized anxiety disorder.

Physical health was evaluated by using the chronicity-variable of the somatic domain of the INTERMED for the elderly (IM-E) interview [30]. Somatic health is assessed by asking, “Which of your physical illnesses have been ascertained over the last 5 years?”. We categorized the participants into three subgroups (1: no chronic disease, 2: one chronic disease, 3: several chronic diseases, ’multimorbidity’).

### Statistical analysis

Participants were included in the analysis if they completed data regarding loneliness and health care costs at the fourth follow-up of the ESTHER study. To characterize the study sample, descriptive statistics were used, while percentages and confidence intervals were calculated to estimate the prevalence of loneliness, stratified by gender and age category. Mean scores and standard deviations of outpatient and inpatient health care use and health care costs were calculated and compared according to the Wilcoxon/ Kruskal-Wallis test.

Multiple regression analyses were performed to predict outpatient health care use by loneliness. Due to the skewed distribution of the response variable (outpatient health care use), generalized linear models with Gamma-distribution were applied. In the first regression model loneliness (0/1), physical health (no chronic disease vs. one vs. >=2), and mental illness (prevalence of depression, GAD and/ or somatization disorder) were simultaneously included as predictors—adjusted for age, marital status, education, gender, and interaction loneliness x gender. As the interaction term loneliness x gender became significant the final prediction models were run separately for women and men. A p-value of < 0.05 was considered significant. For each variable, standardized regression coefficients were built (labeled “β”) to compare the influence between predictor variables directly. Sensitivity analyses were performed by using lognormal regression models.

Statistical analysis was performed using SAS, version 9.4.

## Results

A total of 2525 participants (52.9% female, 47.1% male) were included in the study. They each completed the items regarding loneliness and health care use. Table 1 indicates the demographic characteristics of the study population, separated by the degree of loneliness.

**Table 1:** Demographic characteristics of the study population

Baseline variables	Lonely participants (n = 233)				Less lonely participants (n = 2292)				
	**N**	**(%)**	**95%-CI**	**N**		**(%)**	**95%-CI**	p	
Sex									
Female	173	74.3	68.1; 79.7	1162		50.7	48.6; 52.8	**< 0.001**	
Male	60	25.7	20.3; 31.9	1130		49.3	47.2; 51.4		
Age (years)									
55–64	44	18.9	14.1; 24.5	424		18.5	16.9; 20.2	0.100	
65–74	100	42.9	35.5; 49.5	1138		49.7	47.6; 51.7		
75–84	89	38.2	31.9; 44.8	730		31.8	29.9; 33.8		
Education (years)									
0–9	3	1.3	0.3; 3.7	29		1.3	0.8; 1.8	0.796	
9–10	196	84.1	78.8; 88.6	1869		81.5	79.9; 83.1		
11–12	18	7.7	4.6; 11.9	212		9.3	8.1; 10.4		
>12	16	6.9	4.0; 10.9	182		7.9	6.9; 9.1		
Marital status									
Single	14	6.0	3.3; 9.9	71		3.1	2.4; 3.9	**< 0.001**	
Married	82	35.2	29.1; 41.7	1696		74.0	72.2; 75.8		
Divorced/ widowed	137	58.8	52.2; 65.2	525		22.9	21.2; 24.7		
Physical health									
No chronic disease	60	25.8	20.3; 31.9	755		32.9	31.0; 34.9	0.077	
1 chronic disease	64	27.5	21.8; 33.7	548		23.9	22.2; 25.7		
≥ 2 chronic diseases	109	46.7	40.2; 53.4	989		43.2	41.1; 45.2		
Mental illness									
Yes (Depression/Somatization/ GAD)	56	24.0	18.7; 30.1	154		6.7	5.7; 7.8	**< 0.001**	
No mental disease	177	76.0	69.9; 81.3	2138		93.3	92.2; 94.3		

Total: 2525 participants; CI: confidence interval;

p: Chi2- test of the comparison between lonely and less lonely persons;

Note: significant associations are printed in bold

### Loneliness prevalence and association with age category and gender

At baseline a high degree of loneliness (defined by a total loneliness-score ≥ 7) was measured in 233 older persons (9.2%; CI: 8.1; 10.4). Women were significantly more frequently lonely in comparison to men (13.0% vs. 5.0%; p < .001). While in older women loneliness prevalence was significantly highest in the oldest age category (16.3%; p < .001), only 5.4% of older men (ages 75–84) described loneliness (p = .86). This association was confirmed when adjusting for the higher incidence of widowhood in older women, comparing married persons only (loneliness prevalence in married persons ages 75–84: 6.9% women vs. 2.8% men; p < .001).

### Days of outpatient and inpatient care of lonely persons

Study participants reported an average number of contacts with outpatient care institutions of 7.2 (SD: 7.4; range: 0–68) over a three-month period. Women and men did not differ regarding the number of outpatient contacts (p = .22). However, men had significantly more days of inpatient care compared to women (p = .02). In the gender-specific subgroups, lonely women reported more contact with outpatient care in comparison to less lonely women (p = .01), whereas there was no significant difference between lonely and less lonely men (p = .37). In addition, loneliness was not associated with the number of days of inpatient care for both women and men (see Table 2).

**Table 2:** Number of contacts with health care in lonely and less lonely individuals over a three-month period

	All			Lonely persons			Less lonely persons			
	Mean	SD	Median	Mean	SD	Median	Mean	SD	Median	p
Outpatient care (no.)										
Total	7.2	7.4	5	8.7	8.5	6	7.0	7.3	5	**0.002**
Women	7.4	7.4	5	9.1	8.7	7	7.1	7.1	5	**0.005**
Men	7.0	7.5	5	7.5	7.9	6	7.0	7.5	5	0.368
Inpatient care (no. days)										
Total	1.0	5.2	0	1.6	6.8	0	1.0	5.0	0	0.050
Women	1.0	5.0	0	1.3	5.4	0	1.0	5.0	0	0.088
Men	1.1	5.3	0	2.3	9.9	0	1.0	4.9	0	0.096

p: Wilcoxon/ Kruskal-Wallis test of the comparison between lonely and less lonely persons

Note: significant associations are printed in bold

**Fig. 1:**
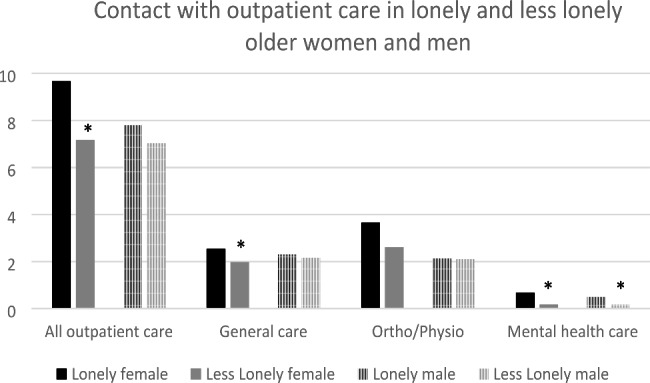
Number of contacts with several sectors of outpatient care (over a three-month period) in lonely and less lonely older women and men. *Significant difference (Wilcoxon/ Kruskal-Wallis test)

An overview of the mean number of contacts with several sectors of outpatient care over the past three months is provided in Fig. 1. In women, contact with general practitioners (p = .002) and mental health care (p = .001) was higher in lonely participants in comparison to less lonely participants. In men, loneliness was not associated with outpatient health care use—except for mental health care, which was significantly and more frequently used by lonely older men (p = .01). In women, 25.4% of the lonely persons were simultaneously diagnosed with mental illness symptoms, compared to 20% of lonely older men. There was no significant difference regarding the frequency of contact with emergency care in lonely and less lonely participants (p = .61) for both genders.

### Average health care costs of lonely participants

Average costs of outpatient care institutions were € 298.1 (SD: 815.4; range: 0-2504.3) over a three-month period. There was no significant difference between older women and men regarding outpatient costs (p = .22) and total costs (p = .48), but men had significantly higher inpatient costs (p = .02) in comparison to women. Lonely women, in comparison to less lonely women, had significantly higher costs regarding outpatient care (p = .007) but not for inpatient care. There was no significant difference in lonely and less lonely older men regarding average costs for outpatient care (p = .120) or inpatient care (p = .09). Please see Table 3.

**Table 3:** Outpatient health care costs of lonely and less lonely individuals

	All			Lonely persons			Less lonely persons			
	Mean	SD	Median	Mean	SD	Median	Mean	SD	Median	p
Outpatient care (€)										
Total	298.1	815.4	144.6	334.0	483.7	179.8	294.5	841.8	142.2	**0.002**
Women	301.3	919.6	148.8	338.1	462.3	181.4	295.8	969.4	144.8	**0.007**
Men	294.5	680.1	140.7	322.1	544.6	167.5	293.1	686.8	139.3	0.196
Inpatient care (€)										
Total	400.2	1861.2	0	539.3	1898.3	0	386.1	1857.3	0	0.051
Women	377.5	1827.3	0	457.9	1784.5	0	365.5	1834.0	0	0.094
Men	425.8	1899.0	0	774.0	2193.3	0	407.3	1881.4	0	0.088
Total costs (€)										
Total	698.3	2070.2	152.1	873.3	1973.5	206.3	680.5	2079.4	148.7	**0.001**
Women	678.8	2077.4	155.7	796.1	1874.0	201.1	661.3	2106.2	151.2	**0.004**
Men	720.3	2062.8	148.1	1096.1	2237.8	229.8	700.3	2052.3	145.1	0.068

p: Wilcoxon/ Kruskal-Wallis test of the comparison between lonely and less lonely persons

Note: significant associations are printed in bold

### Association between loneliness and contact with outpatient care use in adjusted regression models, separated by gender

In the joint regression model the interaction term gender x loneliness became significant (p = .01). Subsequent gender-specific regression analyses showed that loneliness was associated with the number of contacts with outpatient care in women, but not in men. In addition, in women, the number of contacts with outpatient care was positively associated with “being single”, clinical symptoms of mental diseases, and the occurrence of one or more chronic diseases.

Furthermore, in men, the number of contacts with outpatient care was positively associated with the occurrence of clinical symptoms mental diseases, and a chronic disease or multimorbidity.

Please see Table 4 for detailed results of multiple linear regression models, stratified by gender.

**Table 4:** Multiple linear regression analysis (with gamma distribution) for outpatient care use^1^ contact in older women and men

	Women				Men			
Baseline variables	B^2^	SE^3^	CI^4^	p- value	B^2^	SE^3^	CI^4^	p- value
Age (years)								
55–64	0.02	0.06	− 0.10; 0.15	0.70	< 0.01	0.07	− 0.14; 0.14	0.99
65–74 (ref.^5^)								
75–84	0.03	0.05	− 0.08; 0.13	0.59	0.05	0.06	− 0.06; 0.15	0.40
Education (years)								
0–8	< 0.01	0.22	− 0.40; 0.47	0.99	0.22	0.22	− 0.18; 0.67	0.31
9–10 (ref.)								
≥11	− 0.13	0.10	− 0.32; 0.08	0.23	0.07	0.07	− 0.07; 0.21	0.35
11–12	− 0.19	0.10	− 0.38; 0.02	0.06	0.12	0.08	− 0.04; 0.29	0.14
Marital status								
Single	0.27	0.13	**0.03; 0.53**	**0.04**	0.16	0.14	− 0.11; 0.44	0.26
Married (ref.)								
Divorced / widowed	0.04	0.05	− 0.06; 0.14	0.41	0.13	0.08	− 0.02; 0.28	0.09
Physical health								
No chronic disease (ref.)								
1 chronic disease	0.12	0.06	**< 0.01; 0.25**	**0.04**	0.32	0.07	0.18; 0.45	**< 0.001**
≥2 chronic diseases	0.28	0.05	**0.18; 0.39**	**< 0.001**	0.37	0.06	0.25; 0.48	**< 0.001**
Mental disease								
Yes	0.36	0.08	**0.21; 0.52**	**< 0.001**	0.56	0.10	0.37; 0.77	**< 0.001**
No (ref.)								
Loneliness								
High degree	0.17	0.07	**0.03; 0.31**	**0.02**	− 0.10	0.11	− 0.32; 0.14	0.42
Low degree (ref.)								

^1^Outpatient care contact was counted over the past three months; ^2^ estimate; ^3^standard error; ^4^95%-confidence limits; ^5^referent; Note: significant associations are printed in bold

## Discussion

To our knowledge, this is the first study to investigate gender differences regarding the association between loneliness and health care use in older adults. Our data show that older women and men differ in the way they use health care resources and that loneliness is gender-specifically associated with outpatient health care use. For both genders, the occurrence of a mental disease and multimorbidity showed the strongest associations with outpatient health care use. In addition, in older women—but not in older men—outpatient health care use was associated with loneliness. For both genders inpatient care and costs were not related to loneliness.

Our findings correspond with previous studies that show a significantly higher use of inpatient care in older men and a (non-significant) tendency for higher use of outpatient care in older women. Based on other studies that reported a greater openness of women towards seeking help and presenting health problems [31], one could assume that perhaps older men delay seeking assistance or support of outpatient care.

In both genders, impaired mental health and chronic diseases were associated with higher outpatient health care use. This observation is confirmed by current research regarding depression or anxiety and health care costs [32–33]. Mental diseases and loneliness are strongly associated [34–36]. Our own previous research shows that prevalence of depression or generalized anxiety is significantly higher in lonely individuals in comparison to less lonely persons [2, 6].

For the following we would like to emphasize that loneliness in older women only shows a small effect in regard to outpatient health care use compared to the occurrence of mental illness or multimorbidity. However, as loneliness was the primary focus of this study, we will cautiously discuss this result.

From an evolutionary perspective, the pain created by loneliness could alarm persons that something or someone is missing and thereby motivate them to arrange for contact [37]. It can be assumed that women and men react differently to a sudden need for contact. This can be explained by socio-psychological factors. Ko et al. pointed out that older women had more needs regarding care, contact, and emergency services as compared to men [38]. Thus, one hypothesis could be that older women might react earlier to worries about health if they experience a constant thread of loneliness. Another hypothesis is that women are more accustomed to seeking help when they have a problem. A previous study showed that older women in Germany demonstrated a higher level of symptom-reporting in comparison to men [15]. Barsky et al. reported that differences in somatic and visceral perception and a disparate readiness to acknowledge and end discomfort could contribute to this observation [31]. Additionally, Bertakis found that women are more interested in health issues, as is shown by a higher number of preventive contacts [39]. Finally, one should consider that older men—who are possibly more often the recipients of spousal care in this age group [40]- would perhaps address their wives earlier in case of a health problem than vice versa. Nevertheless, it needs to be pointed out that in Germany access to health care is by choice and free of charge due to mandatory health insurance; this makes it easier to overcome any inhibition to contacting health care providers.

Regarding the different domains of outpatient health care, we found that lonely women reported more contact with general care and mental health care, but not with medical-care specialists. This could be explained by a lower inhibition threshold to contact primary care providers in comparison to medical specialists. In men, however, lonely participants showed a higher number of contacts with mental health care only. In both genders the association of loneliness and use of mental health care institutions underlines the strong connection between loneliness and mental diseases on the one hand, but it also underlines the need to properly distinguish between them to find appropriate care.

Similarly, regarding the number of contacts with outpatient health care, we found significantly higher health care costs for outpatient care in lonely older women in comparison to less lonely older women, but not in lonely in comparison to less lonely older men. In several studies the association of gender and health care cost has been described, but with inconsistent results [18]. While some authors describe higher health care cost in older men (increasing with age [41]), others find higher expenditures for health care in older women. This can be explained, perhaps, by a longer life expectancy [42]. However, our data show that it is important to examine both outpatient and inpatient costs gender-specifically to appropriately reflect the various ways women and men deal with their problems in the frame of our health care system. Our 2014 data estimate the mean cost for outpatient care over a three-month period in Germany to be € 298.1 (SD: 815.4). Previous analyses using the same data set found that the mean total annual cost was comparable to those of similar samples of the older population in Germany [21, 43–45].

Based on our cut-off we found a prevalence of loneliness of 9.2%—a prevalence that is in line with current research considering the various methods of measuring loneliness and possible cultural differences [10]. Older women were significantly more frequently lonely in comparison to older men (13% vs. 5%), specifically those who were over 75 years of age. However, data on loneliness prevalence and gender are inconsistent. Several studies, including our own previous data, indicate a preponderance of women regarding loneliness [2, 10]. This could partly be explained by the fact that older women are more frequently lonely because they live longer, are more frequently widowed, and would therefore need more support during older age [12]. However, all these possible interpretations regarding the association between loneliness and health care use in older women and men remain somewhat speculative.

Our study has several limitations: Firstly, we measured loneliness by using only a three-item questionnaire, derived from the GFI. We therefore have no deeper insight into the situation or personality of the persons who reported loneliness, nor do we have any objective data about the participants’ social network. However, the GFI has been proven to be a helpful screening instrument and well-applicable in large epidemiological settings [24]. In previous studies the psychosocial functioning subscale showed good internal consistency and criterion validity [23]. Secondly, selection bias would be possible because only participants who were motivated to join the home visit were included; we also did not include retirement home or nursing home residents. It can be assumed, therefore, that the ESTHER study participants were rather healthy and may therefore use fewer health services [45]. Thirdly, data on health care use were self-reported. In any case, recall bias (possible underreporting) was reduced by recording these data with the help of professional interviewers during the home visit and by investigating over a short period of three months [46].

The particular strength of this study is the large sample size of the study population (n = 2525) and the method of recording data by a comprehensive home visit over several hours conducted by trained study doctors, in addition to questionnaires. The study doctors recorded and assessed chronic diseases and multimorbidity by well-validated measures [29, 30, 47] and listed all outpatient medical contacts of the participants at home. Medical and health-economic data of high quality could be thereby obtained in a large setting of older adults.

## Conclusions

The conclusion of our study is that gender differences are important when investigating health care costs in older persons. In addition, loneliness appears to be associated with elevated outpatient health care contact in older women, but not in men. This emphasizes the need to evaluate loneliness more thoroughly in health care encounters, particularly in older women. Interventions to address and monitor loneliness in older age are needed—not only from a clinical and social perspective, but also from an economic one.

## Data Availability

All data generated or analyzed during this study are included in this published article.

## List of abbreviations

ESTHER: Epidemiologische Studie zu Chancen der Verhütung, Früherkennung und optimierten Therapie chronischer Erkrankungen in der älteren Bevölkerung
GFI: Groningen Frailty Indicator
PHQ: Patient Health Questionnaire
GAD: Generalized Anxiety Disorder- Screener
